# Enhancing Few-Shot Learning in Lightweight Models via Dual-Faceted Knowledge Distillation

**DOI:** 10.3390/s24061815

**Published:** 2024-03-12

**Authors:** Bojun Zhou, Tianyu Cheng, Jiahao Zhao, Chunkai Yan, Ling Jiang, Xinsong Zhang, Juping Gu

**Affiliations:** 1 School of Information Science and Technology, Nantong University, Nantong 226019, China; zhoubj@ntu.edu.cn (B.Z.);; 2 School of Electrical Engineering, Nantong University, Nantong 226019, China; 3 School of Electronic & Information Engineering, Suzhou University of Science and Technology, Suzhou 215009, China

**Keywords:** few-shot classification, knowledge distillation, model compression, distribution calibration

## Abstract

In recent computer vision research, the pursuit of improved classification performance often leads to the adoption of complex, large-scale models. However, the actual deployment of such extensive models poses significant challenges in environments constrained by limited computing power and storage capacity. Consequently, this study is dedicated to addressing these challenges by focusing on innovative methods that enhance the classification performance of lightweight models. We propose a novel method to compress the knowledge learned by a large model into a lightweight one so that the latter can also achieve good performance in few-shot classification tasks. Specifically, we propose a dual-faceted knowledge distillation strategy that combines output-based and intermediate feature-based methods. The output-based method concentrates on distilling knowledge related to base class labels, while the intermediate feature-based approach, augmented by feature error distribution calibration, tackles the potential non-Gaussian nature of feature deviations, thereby boosting the effectiveness of knowledge transfer. Experiments conducted on MiniImageNet, CIFAR-FS, and CUB datasets demonstrate the superior performance of our method over state-of-the-art lightweight models, particularly in five-way one-shot and five-way five-shot tasks.

## 1. Introduction

In today’s digital era, computer vision, as a key branch of AI, is developing rapidly and playing an increasingly important role in many application fields. Especially in the task of image classification, high-performance vision processing systems are crucial to many industries, such as autonomous driving, medical diagnosis, and power inspection [1,2,3]. Recently, researchers have generally adopted complex and large-scale deep learning models in order to improve the classification performance of these systems [4,5,6]. Although these models are designed to have excellent performance, they face a series of challenges when actually deployed in computing resource-constrained environments, such as high computing costs and limited storage space [7].

In view of these limitations, how to improve the performance of lightweight models for classification tasks has become a key research topic in the field of machine learning. Few-shot learning (FSL) is designed to enable models to learn from limited data samples and make accurate predictions [8]. When FSL is applied in some specific scenarios, such as electric mobile inspection or small UAVs, lightweight models are more suitable. However, training lightweight models like large models often encounters the problem of overfitting, causing the actual performance of the model to be far less than expected. To this end, the main goal of this study is to explore a novel method to improve the performance of lightweight models in few-shot classification (FSC) tasks. With this approach, we hope to narrow the performance gap between lightweight and large models on FSC tasks.

**Figure 1:**
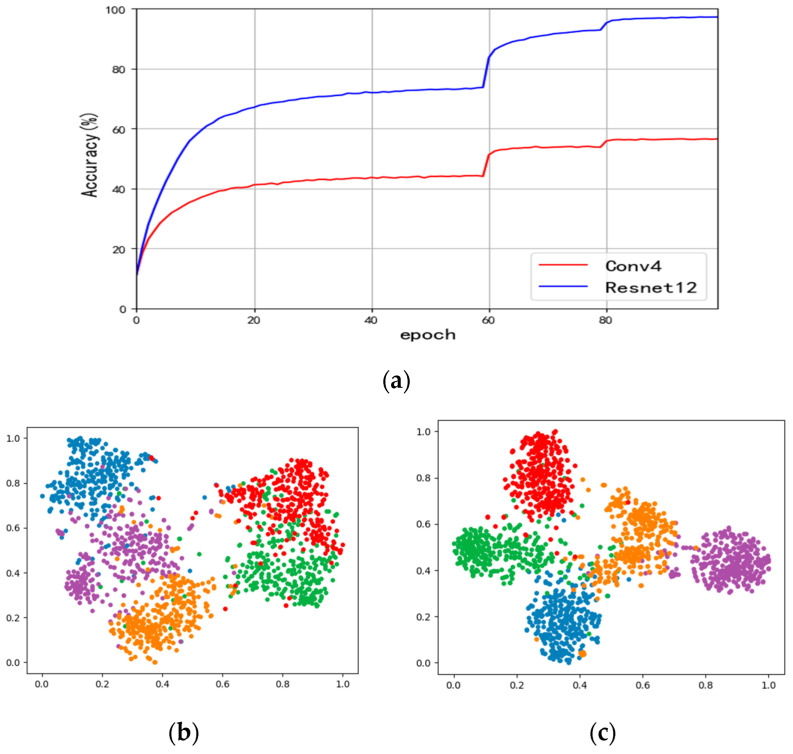
Performance difference between lightweight model (Conv4) and complex model (ResNet12) in few-shot classification tasks. (**a**) The accuracy of classifying base class samples; (**b**) the feature distributions of 5 randomly selected classes post Conv4 processing; (**c**) the feature distributions of 5 randomly selected classes post ResNet12 processing, where each color represents a distinct category of features.

In the field of FSC, Conv4 and ResNet12 represent prevalent neural network architectures [9,10], each offering distinct levels of complexity. Compared to Conv4, ResNet12 is a more complex or larger model. The choice of which model to use may depend on the needs of the particular task as well as the available computational resources. After an in-depth study of the performance differences between a lightweight model (Conv4) and a complex model (ResNet12) in the FSC task, our analysis reveals significant differences in two main areas. First, as shown in Figure 1a, we compare the accuracies of Conv4 and Resnet12 in classifying the base class samples in the pre-training phase. This comparison clearly shows that there is a considerable difference between the two in terms of classification accuracy. The lightweight model does not seem to be able to extract enough information from the input data of the base class to achieve a similar level of performance as the complex model. Second, by visualizing the output features of Conv4 and Resnet12 on the test images, as illustrated in Figure 1b, we further observe that the two models also exhibit significant differences in feature representation. This difference points to a fundamental difference in feature extraction and representation capabilities, providing a key clue to understanding why the lightweight model lags behind in performance.

Based on the above analysis, this study considers lightweight and complex models as student and teacher models, respectively, and conducts a model compression task using a knowledge distillation-based approach, aiming to enhance the performance of lightweight models in FSC tasks. Specifically, this study first applies an output-based knowledge distillation algorithm to enhance the lightweight model’s ability to classify the base class, with the expectation that the student model can mimic and learn the output knowledge of the teacher model. In addition, to further enhance the feature representation capability of the lightweight model, we adapt a distillation method based on feature error distribution calibration. This method works by adjusting the intermediate features of the student model to make it closer to the feature output of the teacher model.

**Figure 2:**
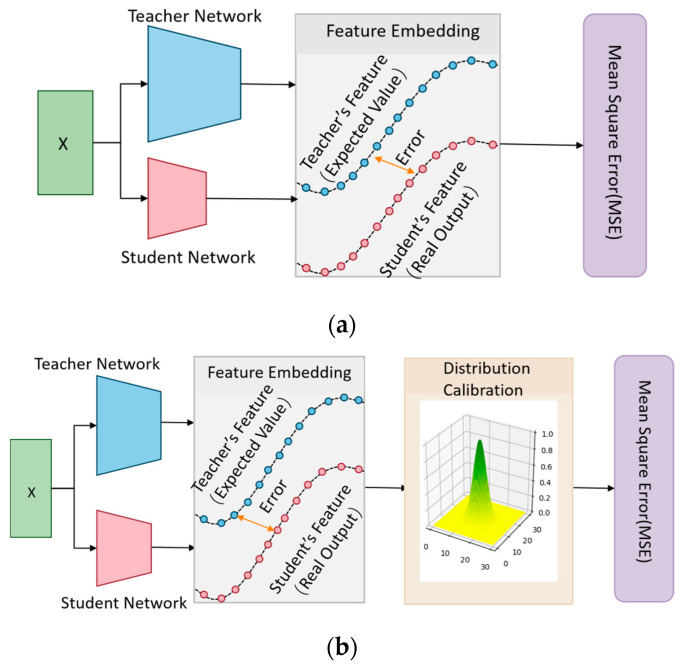
A novel intermediate feature-based knowledge distillation method. (**a**) Previous intermediate feature-based knowledge distillation method; (**b**) a knowledge distillation method based on feature error distribution calibration proposed in this study.

As shown in Figure 2a, previous intermediate feature-based knowledge distillation methods typically take the feature output of the teacher model as the desired output and approximate the teacher model by minimizing the mean square error function of the features. However, the effectiveness of this approach depends on the assumption that the data follow a Gaussian distribution, as the mean square error loss function is a natural choice for maximum likelihood estimation under a Gaussian distribution (proof is provided in Section 3.3.1). If the feature errors do not follow a Gaussian distribution, relying only on the mean square error may result in the training of the student model deviating from the optimum. With this in mind, this study proposes a novel knowledge distillation strategy. As shown in Figure 2b, this strategy first applies Gaussian calibration to the distribution of feature errors between the teacher and student models, and then constructs the mean square error loss function. This approach helps the student model mimic the intermediate features of the teacher model more accurately, thus improving its classification performance.

In summary, the contributions of this paper are as follows:

(1) We investigate the application of the dual-faceted knowledge distillation method to the task of few-shot model compression to enhance the performance of lightweight models.
(2) We develop a novel method for calibrating feature error distribution that significantly enhances the performance of feature-based knowledge distillation. Furthermore, we provide theoretical proof to substantiate our proposed method, offering valuable insights and establishing a robust framework for future research in this field.
(3) We demonstrate the effectiveness of our proposed method by validating it on three benchmark datasets. Our proposed method outperforms all other methods and achieves the best performance.

## 2. Related Work

### 2.1. Few-Shot Classification

Depending on the learning paradigm used to acquire prior knowledge, most of the current few-shot classification methods can be categorized into meta-learning-based methods and transfer learning-based methods [11]. Meta-learning, also known as learn to learn [12], is the dominant learning paradigm for solving few-shot image classification tasks. This learning strategy involves randomly selecting a number of few-shot classification tasks, using epoch training to acquire the meta-knowledge implicit in these tasks, and quickly processing unseen tasks based on the meta-knowledge [13]. Depending on the type of meta-knowledge learned, these methods are further classified into optimization-based methods and metric-based methods. Compared to the former, metric-based methods generally have better classification performance. Learning to obtain good features for distinguishing different classes of samples is the key to achieving good performance for these methods. For example, PN+rot [14] improved the generalization performance of metric models by adding the rotation prediction task from self-supervised learning to the meta-learning process. Recently, refs. [15,16] have made improvements in improving the generalization performance of prototype networks, with the former focusing on normalizing the prototypes computed based on the support samples in each sub-task, while the latter adopted the approach of estimating in regenerated Hilbert space to obtain the relative prototypes of each class of support samples and introducing the Tikhonov regularization technique in the training process.

Recent research has shown that in transfer learning, using a whole base class dataset to pre-train the model and fine-tune the classifier using a small number of samples can achieve comparable performance to meta-learning. In this context, early work [17] focused on pre-training convolutional neural networks using the cross-entropy loss function, providing a powerful baseline for the task of FSC. In this classification framework, how to obtain an effective feature representation in the pre-training phase is crucial to improving the performance of FSC. In order to enhance the generalization ability of the traditional cross-entropy loss function, Neg-Cosine [18] and S2M2 [19] introduced non-negative-spaced cosine loss function and streaming mixing techniques, respectively. These methods are proposed to enhance the classification accuracy by improving the loss function and enhancing the richness of the feature representation. In addition, self-supervised tasks have been shown to enhance the generalization performance of FSC models in a transfer learning framework. For instance, IE [20] augmented the model by integrating self-supervised tasks such as rotation prediction and instance discrimination, thereby enhancing the model’s invariance and equivariance to variations in input. Meanwhile, CCF [21] introduced the task of generating features during the pre-training process to further enhance the model’s representational capabilities.

Considering recent advances in the task of FSC, a common trend can be observed: whether based on meta-learning or transfer-learning approaches, scholars generally tend to adopt complex models to enhance the capability of feature representation. However, these complexity models tend to consume a large number of computational resources in practical applications and also lead to higher time delays. These shortcomings limit the application of these methods in resource-constrained real-world scenarios requiring fast responses. In view of this, this paper proposes a few-shot model compression method based on a dual-knowledge distillation strategy that has important practical significance and research value.

### 2.2. Knowledge Distillation

Knowledge distillation is a very effective method of model compression that employs a high-capacity teacher model to instruct a compact student model so that the student has comparable performance to the former. The inter-model knowledge distillation method was first proposed by Hinton [22]. In order to better utilize the knowledge information contained in the teacher’s model, recent work has focused more on how to better extract the feature knowledge implicit in the middle layer of the network. For example, FitNet [23] directly minimizes the L2 paradigm between the feature outputs of the corresponding layers between the student network and the teacher network. AT [24] proposes to use the spatial attention of the features in the implicit layer of the teacher network as the knowledge and instructs students to mimic the teacher’s attentional feature maps. SP [25] and RKD [26] compute the relationship matrix between image features in the student model and the teacher model, enabling the student to better learn the teacher’s relational knowledge. PKT [27] and NST [28] align the overall distribution of the student and teacher output features using the mutual information scatter measure and maximum mean difference, respectively.

Several recent research works have also introduced knowledge distillation techniques to FSC tasks. For example, RFS [29] and SKD [30] utilize a network regeneration strategy to obtain a more robust feature extraction model through continuous evolution between multiple generations. PAL [31] expects the student model to align to the teacher model in terms of output logical values and implicit features, respectively. BML [32] utilizes the complementary nature of meta-learning and transfer learning to train the student model separately while using deep mutual learning techniques to facilitate collaborative learning among student models during the training process. In the above work, both the student and the teacher model are self-distilled using complex models with the same structure. Contrary to the aforementioned approaches, this study employs knowledge distillation as a strategy for transferring knowledge from a complex model to a lightweight counterpart, with the primary goal of enhancing the performance of the lightweight model.

## 3. Main Approach

### 3.1. Overview

According to the process setup of the few-shot image classification task [33], the given image dataset is randomly partitioned into three subsets, i.e., the base class dataset $D_{b}$, the validation dataset $D_{val}$, and the novel class dataset $D_{n}$. These three subsets, which have different class labels, are used for the training of the model, the validation of the model, and the few-shot testing, respectively. In the model validation and few-shot testing phases, several N-way K-shot classification tasks need to be constructed, each of which contains a collection of support samples and a collection of query samples. K samples from N categories are randomly selected from $D_{val}$ or $D_{n}$ to construct the support set $D_{s}={\left\{\left(x_{k},y_{k}\right)\right\}}_{k=1}^{NK}$, where $x_{k}$ denotes the *k*-th image and $y_{k}$ is its corresponding category label. The remaining samples from N categories form the query set, denoted as $D_{q}={\left\{\left(x_{q},y_{q}\right)\right\}}_{q=1}^{Q}$, where $x_{q}$ denotes the *q*-th query sample.

**Figure 3:**
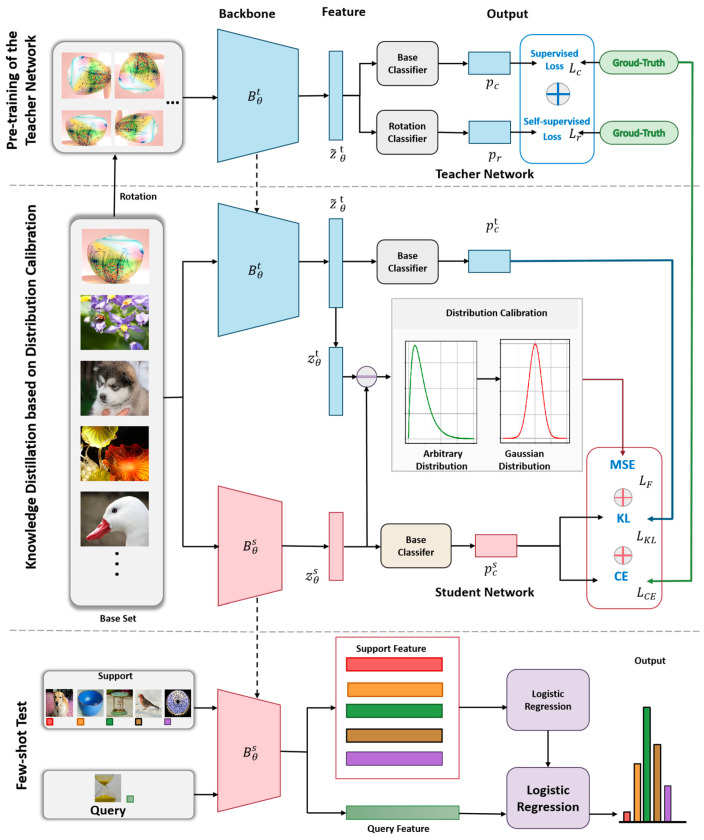
The framework diagram of our method. It presents a framework for model compression in few-shot image classification using dual-faceted knowledge distillation. Initially, a teacher network is trained with combined supervised and self-supervised losses. Knowledge is then distilled to a lightweight student model through two strategies focusing on output and feature knowledge. Finally, the student model is evaluated on FSC tasks, demonstrating its ability to generalize effectively with limited data.

As shown in Figure 3, the methodological framework proposed in this paper is divided into three main phases. In the first phase, we train a well-performing teacher network (Resnet12) on $D_{b}$ using a weighted sum of cross-entropy loss and self-supervised loss. This step aims to obtain robust feature representations by combining standard supervised learning and self-supervised learning. The second stage involves the use of an innovative dual-knowledge distillation strategy that aims to efficiently migrate the output knowledge and intermediate layer feature knowledge from the teacher model to the student model (Conv4). 

The backbone of our proposed model consists of a four-layer convolutional network, Conv4. Each layer comprises a sequence of a convolutional layer, batch normalization, and a ReLU activation function. The first three layers are followed by a max-pooling layer to reduce the dimensionality of the features. Contrary to the preceding layers, the final convolutional layer is directly followed by an adaptive average pooling layer, which reduces the spatial dimensions of the feature maps to 1 × 1, facilitating the extraction of the feature vectors.

In this way, we accomplish the task of compressing the few-shot image classification model, aiming to reduce the complexity of the model while minimizing the performance gap between the two. In the third phase, we deploy the trained lightweight student network on $D_{n}$ for a few-shot image classification test. This phase mainly verifies the effectiveness of the lightweight model after model compression on the new task. The key steps and detailed flow of this method will be described and discussed in detail in the remainder of this section.

### 3.2. Teacher Model Construction

Recent studies have shown that self-supervised learning effectively improves the generalization performance of transfer-learning models in FSC tasks [34]. Based on this, in this paper, we use a linear combination of cross-entropy and self-supervised loss function to pre-train the teacher network model and select rotation prediction as the self-supervised learning task. The structure of the teacher model mainly consists of a backbone network $B_{\theta }^{t}$, two linear classifiers, and their corresponding Softmax classification layers, where $\theta_{t}$ represents the parameters of the backbone network. A linear classifier $C_{b}\left(\cdot \right)$ is used for the prediction of the base classes with a parameter matrix of $W_{b}={\left\{w_{c}\right\}}_{c=1}^{C}$. Another linear classifier $C_{r}\left(\cdot \right)$ is used for the prediction of the rotated classes, and its parameter matrix is $W_{r}={\left\{w_{r}\right\}}_{r=1}^{R}$. $w_{c},w_{r}\in R^{d}$ are *d*-dimensional weight vectors, and C and R are denoted as the number of base categories and rotated categories, respectively.

In the base class dataset, a number of batches of *L* images are randomly selected, and each image is rotated by 0°, 90°, 180°, and 270° and labeled with its rotation class $o_{i}=[0,1,2,3]$. Each image is inputted into the teacher network, and the backbone of the network maps it into the d-dimensional feature space. The feature can be represented as:


(1) $$z_{i}^{t}=B_{\theta_{i}}(x_{i})\in R^{d}$$

Subsequently, the probabilistic predicted values $p_{ic}$ and $p_{ir}$ are obtained for the base class and the rotated class through the two linear classifiers and the corresponding Softmax layers, respectively, which are computed as follows:

(2) $$\begin{array}{l} p_{ic}=\frac{e^{w_{c}z_{i}^{t}}}{\sum_{c=1}^{C}e^{w_{c}z_{i}^{t}}}\\ p_{ir}=\frac{e^{w_{r}z_{i}^{t}}}{\sum_{r=1}^{R}e^{w_{r}z_{i}^{t}}} \end{array}.$$

Then, the cross-entropy loss function between the base class predicted values and the rotated predicted values and their corresponding ground truth values are calculated as:

(3) $$\begin{array}{l} L_{c}(\theta_{t},W_{b})=\sum_{i=1}^{RL}\sum_{c=1}^{C}y_{ic}logp_{ij}\\ L_{r}(\theta_{t},W_{r})=\sum_{i=1}^{RL}\sum_{r=1}^{R}o_{ir}logp_{ir} \end{array}.$$

The total loss function for pre-training the teacher network is denoted as:

(4) $$L_{t}\left(\theta_{t},W_{b},W_{r}\right)=L_{c}\left(\theta_{t},W_{b}\right)+\lambda L_{r}\left(\theta_{t},W_{r}\right),$$

where *λ* is the adjustable weight parameter. Utilizing Equation (4), the pre-training of the teacher network is accomplished through the application of the gradient descent algorithm.

### 3.3. Dual-Faceted Knowledge Distillation Strategy

#### 3.3.1. Theoretical Foundation

Since the student model has a very small capacity to handle complex self-supervised tasks, the student model mainly learns the base class classification task from the teacher model. The network structure of the student model mainly consists of a backbone $B_{\theta_{s}}$, a linear classifier, and a corresponding Softmax classification layer, where $\theta_{s}$ represents the parameters of the backbone of the model. The linear classifier $C_{s}\left(\cdot \right)$ is used for the prediction of the base classes, and its parameter matrix is $W_{s}={\left\{w_{c}^{s}\right\}}_{c=1}^{C}$, where $w_{c}^{s}\in R^{m}$ is an m-dimensional classification weight vector.

A number L of image batches are randomly selected from the base class data set, where the corresponding base class label of the *i*-th image $x_{i}$ is $y_{i}$. Each image is input to both the teacher network and the student network. The teacher’s and student’s backbone networks are mapped to *d*- and *m*-dimensional feature spaces, respectively, to obtain features represented as follows:

(5) $$\begin{array}{l} {\hat{z}}_{i}^{t}=B_{\theta_{t}}\left(x_{i}\right)\in R^{d}\\ z_{i}^{s}=B_{\theta_{s}}\left(x_{i}\right)\in R^{m} \end{array}.$$

Since the dimensions between the output features of the student and the teacher network are not the same, direct matching cannot be performed. For this reason, a linear matching layer $P\left(\cdot \right)$ is introduced, which maps the feature output of the teacher network to the m-dimensional feature space. At this time, the features of the teacher network are represented as follows:

(6) $$z_{i}^{t}=P\left({\hat{z}}_{i}^{t}\right)\in R^{m}.$$

At this point, the error between the transformed teacher network features and the student network features is computed to obtain the m-dimensional feature error variable *e*, whose *i*-th observation is denoted as:

(7) $$e_{i}=z_{i}^{t}-z_{i}^{s}.$$

**Proposition** **1.** *For a given image x, suppose that the intermediate feature output variable of the student network is *$z^{s}$*with L observations *${\left\{z_{i}^{s}\right\}}_{i=1}^{L}$*and the intermediate feature output variable of the teacher network is *$z^{t}$*with L observations *${\left\{z_{i}^{t}\right\}}_{i=1}^{L}$. *The intermediate feature-based knowledge distillation process takes the feature output value *$z^{t}$*of the teacher network as the target variable, and the student network makes the feature output value *$z^{s}$*approximate to the target variable by optimizing the model parameters. When the error variable *e *between the output features of the teacher network and the student network obeys a Gaussian distribution, the mean square error function is the optimal loss function.*

**Proof of Proposition** **1.** The knowledge distillation process for intermediate features can be described using the joint probability density function $p\left(x,\;e\right)$. By applying conditional probability distribution, this function can be further expressed as:

(8) p(x,e)=p(e|x)p(x).

For a set of *L* independently and identically distributed training samples, the likelihood function is:

(9) $$L\left(\theta_{s}\right)=\prod_{i=1}^{L}p\left(e_{i},x_{i}\right)=\prod_{i=1}^{L}p\left(e_{i}|x_{i}\right)p\left(x_{i}\right).$$

Taking the logarithm of both sides of Equation (9) yields:

(10) $$\ln L\left(\theta_{s}\right)=\sum_{i=1}^{L}\ln p\left(e_{i}|x_{i}\right)+\sum_{i=1}^{L}\ln p\left(x_{i}\right).$$

To maximize the likelihood function, the objective function $L_{F}\left(\theta_{s}\right)$ is defined as:

(11) $$L_{F}\left(\theta_{s}\right)=-\ln L\left(\theta_{s}\right)=-\sum_{i=1}^{L}\ln p\left(e_{i}|x_{i}\right)-\sum_{i=1}^{L}\ln p\left(x_{i}\right).$$

In Equation (11), the second term is independent of the student network and the objective function is simplified to:

(12) $$L_{F}\left(\theta_{s}\right)=-\sum_{i=1}^{L}\ln p\left(e_{i}|x_{i}\right).$$

Assuming that the errors $e\in R^{m}$ follow a Gaussian distribution with mean 0 and variance $\sigma^{2}$, $p\left(e|x\right)$ is determined as:

(13) $$p\left(e|x\right)=\frac{1}{{\left(2\pi \right)}^{m/2}\sigma^{m}}\exp\left\{-\frac{e^{T}e}{2\sigma^{2}}\right\}.$$

Substituting Equation (13) into the objective Function (12), we get:

(14) $$\begin{array}{l} L_{F}\left(\theta_{s}\right)=-\sum_{i=1}^{L}\ln\frac{1}{{\left(2\pi \right)}^{m/2}\sigma^{m}}\exp\left\{-\frac{{e_{i}}^{T}e_{i}}{2\sigma^{2}}\right\}\\ \;\;=\frac{1}{2\sigma^{2}}\sum_{i=1}^{L}{e_{i}}^{T}e_{i}+\frac{Lm}{2}\ln2\pi +Lm\ln\sigma \end{array}.$$

In Equation (14), the second and third terms are independent of the student network, and since the covariance $\sigma^{2}$ is a constant, the objective function can be further simplified to:

(15) $$L_{F}\left(\theta_{s}\right)=\frac{1}{2}\sum_{i=1}^{L}{e_{i}}^{T}e_{i}.$$

This demonstrates that under the assumption of Gaussian-distributed errors, the optimal objective function for knowledge distillation of intermediate features is the mean squared error of those features. □

#### 3.3.2. Intermediate Feature-Based Distillation

According to Proposition 1, it can be seen that in intermediate feature-based knowledge distillation, the model can achieve the optimum by using the mean square error function as the objective function only when the error variable between the output features of the teacher model and the student model obeys a Gaussian distribution. However, in practice, the probability density distribution of the feature errors between the teacher model and the student model does not necessarily satisfy the Gaussian distribution, and when the mean square error function is used to establish the objective function, the result will deviate from the true optimum.

In order to solve the problem, this paper proposes a novel knowledge distillation algorithm based on feature error distribution calibration. Firstly, the probability density distribution of the feature error variable between the teacher model features and the student model features is calibrated to a Gaussian distribution. In this paper, Tukey Ladder of Powers transformation [35] is used to correct the feature error variables in equalization (16) and scale to unit length, which is calculated as follows:

(16) $${\tilde{e}}_{i}=\{\begin{array}{c} \frac{{\left(e_{i}+\varepsilon \right)}^{\beta }}{∥{\left(e_{i}+\varepsilon \right)}^{\beta }{∥}_{2}},\beta \neq 0\\ \frac{\log\left(e_{i}+\varepsilon \right)}{∥{\left(e_{i}+\varepsilon \right)}^{\beta }{∥}_{2}},\beta =0 \end{array},$$

where *ε* is a very small positive real number and *β* is an adjustable parameter, the skewness of the distribution can be controlled by adjusting *β* during the experimental process, and the distribution of the feature error variables can be made close to the Gaussian distribution.

After distribution calibration, the mean square error loss function based on the intermediate features is calculated as:

(17) $$L_{F}\left(\theta_{s}\right)=\frac{1}{2}\sum_{i=1}^{L}{{\tilde{e}}_{i}}^{T}{\tilde{e}}_{i}.$$

#### 3.3.3. Output-Based Distillation

For the features $z^{t}$ and $z^{s}$ obtained from the teacher and student models, respectively, they are each passed through their respective base class predictive linear classifiers followed by the corresponding Softmax layers. This process yields the probability outputs of the base classes for the student network, calculated as follows:

(18) $$p_{ic}^{s}=\frac{e^{w_{c}^{s}z_{i}^{s}}}{\sum_{c=1}^{C}e^{w_{c}^{s}z_{i}^{s}}}.$$

At the same time, the soft target for the probability output value of the base classes in the teacher model is obtained as:

(19) $$p_{ic}^{t}=\frac{e^{\tau w_{c}{\hat{z}}_{i}^{t}}}{\sum_{c=1}^{C}e^{\tau w_{c}{\hat{z}}_{i}^{t}}},$$

where *τ* represents the temperature parameter. Consequently, we employ the classical knowledge distillation algorithm, enabling the student model to learn the soft target output by the teacher network. The calculation formula for the Kullback–Leibler (KL) divergence between the soft target output by the teacher model and the output information of the student model is as follows:

(20) $$L_{KL}\left(\theta_{s},W_{s}\right)=-\sum_{i=1}^{L}\sum_{c=1}^{C}p_{ic}^{s}\log\frac{p_{ic}^{s}}{p_{ic}^{t}}.$$

### 3.4. Model Evaluation and Testing

To enhance the base class prediction capabilities of the student model, this study also introduces the cross-entropy loss function to reduce the deviation between the predicted values and ground truth. The calculation formula for the cross-entropy loss function is:

(21) $$L_{CE}\left(\theta_{s},W_{s}\right)=-\sum_{i=1}^{L}\sum_{c=1}^{C}y_{ic}p_{ic}^{s}.$$

As a result, the overall loss function for knowledge distillation in the student model is calculated as:

(22) $$L_{s}\left(\theta_{s},W_{s}\right)=L_{CE}\left(\theta_{s},W_{s}\right)+\alpha_{1}L_{KL}\left(\theta_{s},W_{s}\right)+\alpha_{2}L_{F}\left(\theta_{s}\right),$$

where *α*_1_ and *α*_2_ are balancing coefficients, controlling the importance of each loss term in the loss function. Based on Equation (22), the optimization of parameters in the student model is completed using the gradient descent algorithm.

After the model training is completed, the base class classifier in the student network is removed, and the parameters $\theta_{s}$ in the backbone network $B_{\theta_{s}}$ are fixed. The features of images from the support set and the query set are extracted by feeding them into the student network. The feature representations of the k-th support sample and the *q*-th query sample are as follows:

(23) $$\begin{array}{l} z_{k}=B_{\theta_{s}}\left(x_{k}\right)\in R^{t}\\ z_{q}=B_{\theta_{s}}\left(x_{q}\right)\in R^{t} \end{array}.$$

Using $z_{k}$ and the corresponding label $y_{k}$, a logistic regression classifier $g_{\emptyset }(\cdot )$ with parameter ∅ is trained. The classification prediction for the *q*-th query sample is represented as:

(24) $${\hat{y}}_{q}=g_{\phi }\left(z_{q}\right).$$

As detailed in Section 3, the implementation process of this method is displayed in Algorithm 1.
**Algorithm 1 Implementation: Few-Shot Model Compression Algorithm.****Input:** Base class dataset $D_{b}$, validation dataset $D_{val}$, and the novel class dataset $D_{n}$,   the teacher network $\left\{B_{\theta }^{t}\left(\cdot \right),C_{b}\left(\cdot \right),C_{r}\left(\cdot \right)\right\}$, the student network $\left\{B_{\theta }^{s}\left(\cdot \right),C_{s}\left(\cdot \right)\right\}$,   temperature parameter τ, hyperparameter $\alpha_{1}$, $\alpha_{2}$, β.**Output:** The predicted value ${\hat{y}}_{q}$ of query samples in $D_{n}$**Stage 1: Teacher network pre-training****While** epoch ≤ maximum number of the iteration   A batch of images is randomly selected from $D_{b}$.   Images are fed into the backbone of the teacher network to extract the feature.   Obtain the base class and rotation class probability values.   Pre-train the teacher network according to Equation (4).**Stage 2: Few-shot model compression****While** epoch ≤ maximum number of the iteration   A batch of images is randomly selected from $D_{b}$.   The image is separately fed into the backbone of the teacher and the student networks to extract features.   Obtain the base class probability values from the teacher network and the student network, respectively.   Calibrate the feature error distribution between the student network and the teacher network according to Equation (16).   Calculate the knowledge distillation loss function for intermediate features according to Equation (17).   Calculate the KL divergence-based loss function between the predicted output values of the student network and the teacher network according to Equation (20).   Calculate the cross-entropy loss function of the student network according to Equation (21).   Train the student network according to Equation (22).**Stage 3: Few-shot model testing****While** epoch ≤ maximum number of the iteration   Images from $D_{n}$ are processed through the feature extractor to obtain the feature.   Train classifier $g_{\emptyset }(\cdot )$ for the novel classes.   Test on the query set from $D_{n}$.

## 4. Experiments

### 4.1. Dataset

In this study, we conduct comprehensive experiments to evaluate the performance of our proposed method on three benchmark databases for few-shot image classification. MiniImageNet [36] is a subset derived from the larger ImageNet dataset. CIFAR-FS [37] is constructed based on the CIFAR100 dataset. Caltech-UCSD Bird-2002011 (CUB) [38] is the most widely used benchmark in the domain of fine-grained image classification.

### 4.2. Experimental Setup

In this study, all experiments were implemented on a Dell workstation equipped with an NVIDIA 3090Ti GPU at Nantong University, Nantong, China. The software used for these experiments was Pytorch version 1.7.0. For the few-shot image classification tasks, the most commonly used complex model is ResNet12, which we adopted as the teacher model in our method. The most frequently utilized lightweight model in this domain is the four-layer convolutional neural network (Conv4), selected as the student model in our approach. Each convolutional layer of this model contains 64 filters. During the training phase, the stochastic gradient descent (SGD) optimizer was employed, with the momentum set to 0.9 and weight decay set to 5 × 10^−4^. The models were trained for 160 epochs, with an initial learning rate of 0.025. The learning rate was reduced to one-tenth of its value every 30 epochs after the first 70 epochs. For the calculation of the soft targets in the teacher network’s output, the temperature coefficient was set to 0.25. During the testing phase, experiments were conducted in both five-way one-shot and five-way five-shot tasks. For this purpose, 2000 random classification subtasks were generated in the novel class dataset. In each subtask, 15 images per class were randomly selected as query images for testing. The performance was evaluated based on the average accuracy across all subtasks, with the standard deviation of the accuracy given under a 95% confidence interval.

### 4.3. Methodological Validation

#### 4.3.1. Model Compression Efficacy

In pursuit of enhancing the few-shot image classification capabilities of lightweight models, this study introduces a novel model compression method employing dual-faceted knowledge distillation. To validate the effectiveness of this innovative approach, we conducted a series of evaluations comparing the performance of standard lightweight models (referred to as “baseline”), trained solely using cross-entropy loss, with models augmented by our novel compression technique (referred to as “model compression”, or MC). Table 1 presents the comparative classification accuracy of both the baseline and the MC strategy in five-way one-shot and five-way five-shot classification tasks on three datasets: MiniImageNet, CIFAR-FS, and CUB.

The results presented in Table 1 indicate that our model compression method significantly outperforms the baseline in classification accuracy for both five-way one-shot and five-way five-shot tasks across all benchmark datasets. For MiniImageNet, the MC method improves upon the baseline by 4.63 percentage points in the one-shot task and by 1.04 percentage points in the five-shot task. In the CIFAR-FS dataset, the performance gains are 3.19 percentage points for the one-shot and 2.84 percentage points for the five-shot tasks. Likewise, on CUB, MC records increases of 5.15 percentage points for the one-shot and 6.1 percentage points for the five-shot tasks. These results, further detailed in Section 3, underscore the effectiveness of our proposed compression method in enhancing the few-shot classification capabilities of lightweight models.

#### 4.3.2. Feature Error Distribution Calibration

In this research, we introduce a novel knowledge distillation algorithm enhanced by feature error distribution calibration. This section examines the impact of the calibration phase on the algorithm’s performance. We refer to the algorithm without calibration as KD-Plain and the one with calibration as KD-Improved. Table 2 illustrates the accuracy of both KD-Plain and KD-Improved on the MiniImageNet, CIFAR-FS, and CUB datasets for five-way one-shot and five-way five-shot tasks. The results, as shown in Table 2, consistently indicate that KD-Improved outperforms KD-Plain across all datasets and tasks.

The efficacy of our knowledge distillation algorithm, which includes the calibration step, is thus effectively demonstrated. This step addresses the common issue in distillation algorithms that rely on intermediate features, which is the assumption that feature deviations follow a Gaussian distribution. By calibrating these deviations towards a Gaussian model, our approach allows the mean squared error loss to work more effectively. Further details on the calibration process and its optimization criteria have been discussed in Section 3.3.

### 4.4. Comparative Studies

#### 4.4.1. Comparison with Classical Knowledge Distillation Approaches

In this research, we benchmark our novel knowledge distillation algorithm, which employs feature error distribution calibration, against classical methods in the field within the context of model compression for few-shot image classification tasks. We categorized these methods into two groups: those utilizing output knowledge, such as Hinton’s knowledge distillation (HKD) [22], and those based on intermediate feature knowledge, such as FitNet [23], AT [24], SP [25], RKD [26], PKT [27], and NST [28]. The classification results of these methods are detailed in Table 3.

Table 3 demonstrates that our method, which leverages both class intermediate feature knowledge and class output, consistently outperforms the traditional approaches on the MiniImageNet, CIFAR-FS, and CUB datasets. Notably, on CUB, our method shows a marked improvement over the best-performing traditional knowledge distillation technique, with an increase of 1.64% in one-shot and 1.2% in five-shot tasks. Our approach’s enhancements are also evident when directly compared to FitNet and AT, where it exhibits superior accuracy, surpassing AT by 2.02% in one-shot and by 1.74% in five-shot tasks on CUB. Furthermore, our algorithm outperforms SP and RKD, which do not account for the error distribution in relational features, as well as PKT and NST, which may neglect rich sample information with their simpler global relationship models. Our method achieves the largest performance gains over NST on CUB, with improvements of 1.85% in one-shot and of 1.25% in five-shot tasks. These results confirm the efficacy of our algorithm, particularly its feature error distribution correction component, in improving the few-shot image classification performance of lightweight models in compression scenarios.

#### 4.4.2. Comparison with Other Methods

Our proposed method is compared with recent state-of-the-art (SOTA) lightweight model-based methods on MiniImageNet, CIFAR-FS, FC100, and CUB datasets. Consistent with the classification standards in related works, these methods are categorized into meta-learning and transfer learning paradigms. The comparative results of our method against these SOTA methods are detailed in Table 4.

Table 4 indicates that (1) on MiniImageNet, CIFAR-FS, and CUB datasets, our method outperforms all SOTA methods based on lightweight models in both five-way one-shot and five-way five-shot classification tasks. (2) On MiniImageNet, among all the meta-learning-based SOTA methods, HGNN exhibits the best performance in few-shot image classification. Compared to HGNN, our method still achieves higher accuracy by 2.36% and 0.82% in the one-shot and five-shot tasks, respectively. Among the transfer learning-based SOTA methods, CGCS shows the best performance. In comparison to CGCS, our method also excels by 2.46% and 1.18% in the one-shot and five-shot tasks, respectively. (3) On CIFAR-FS, PSST stands out as the best performer. Our method surpasses PSST by 2.65% and 0.56% in the one-shot and five-shot tasks, respectively. (4) On CUB, our method achieves the best results among lightweight models in both one-shot and five-way tasks. It surpasses HGNN by 0.9% in the one-shot task and CGCS by 0.9% in the same task. These results demonstrate the effectiveness of our method in enhancing few-shot image classification capabilities of lightweight models on both generic and fine-grained classification datasets.

### 4.5. Detailed Analysis

#### 4.5.1. Parameter Analysis

In Section 3.4, we detail the optimization of the student model’s comprehensive loss function, outlined in Equation (22), with a particular focus on the coefficients α1 and α2, which are crucial to the model’s performance. We embarked on an extensive grid search to ascertain the optimal values for these coefficients, exploring a parameter range from 0.1 to 1.0 for both α1 and α2. This methodical search was designed to not only individually assess the impact of each coefficient but also to understand their combined effect on the classification performance through a joint analysis.

**Figure 4:**
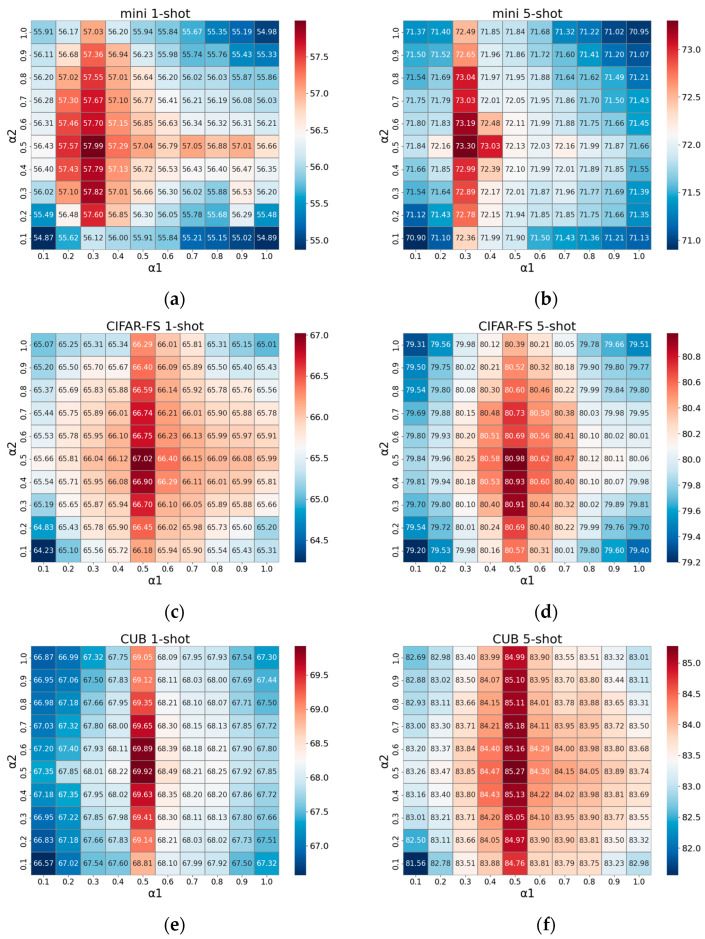
Heatmap visualization of α1 and α2 grid search on three datasets. (**a**) Grid search results on MiniImageNet in 1-shot task; (**b**) grid search results on MiniImageNet in 5-shot task; (**c**) grid search results on CIFAR-FS in 1-shot task; (**d**) grid search results on CIFAR-FS in 5-shot task; (**e**) grid search results on CUB in 1-shot task; (**f**) grid search results on CUB in 5-shot task.

The results of this combined parameter search are visually summarized in Figure 4, where we employ heatmaps to illustrate the classification accuracy across the spectrum of α1 and α2 values. These heatmaps clearly highlight the regions of optimal performance, identified by the deepest red squares, representing the most effective parameter combinations for our model. Although there is a slight variation in the optimal values of α1 and α2 across different datasets, the patterns observed for one-shot and five-shot settings are consistent, reinforcing the reliability of our findings. Specifically, the optimal settings on MiniImageNet are identified as α1 = 0.3 and α2 = 0.5, whereas for CIFAR-FS and CUB, the best-performing values are both α1 and α2 at 0.5.

Furthermore, we extended our exploration of α1 and α2 beyond the standard [0, 1] range for the rigor of the experimental results. However, this additional analysis did not reveal any superior results, confirming that the optimal parameter values are indeed within the initial search range. 

**Figure 5:**
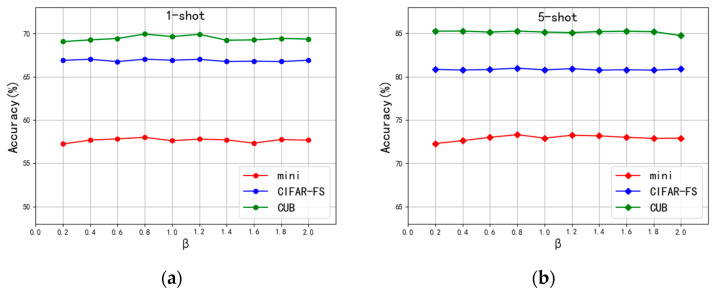
Test experiments under different values of *β* on three datasets: (**a**) 1-shot test accuracy under different values of *β*; (**b**) 5-shot test accuracy under different values of *β*.

Additionally, Equation (16) from Section 3.3.2 introduces *β*, which modulates the skewness in Gaussian feature error calibration. Testing *β* values in the [0, 2] range by 0.2 steps, we found, as demonstrated in Figure 5, that 0.8 delivers optimal performance across all datasets and tasks. Consequently, *β* was set to 0.8 for all further analyses.

#### 4.5.2. Visualization Analysis

**Figure 6:**
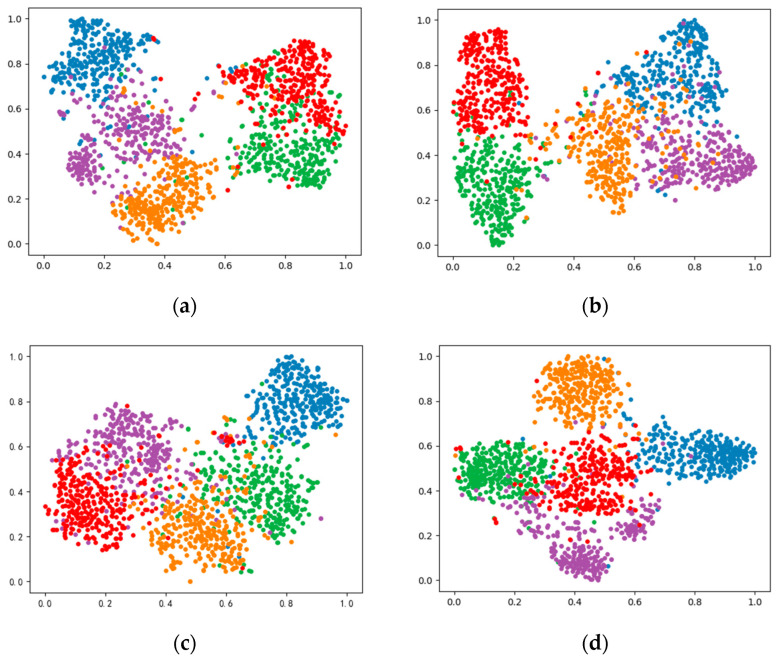
The t-SNE visualization of feature distributions on MiniImageNet and CIFAR-FS. (**a**) The feature distributions of 5 randomly selected novel classes from MiniImageNet by the baseline method; (**b**) the feature distributions of 5 randomly selected novel classes from MiniImageNet by our method; (**c**) the feature distributions of 5 randomly selected novel classes from CIFAR-FS by the baseline method; (**d**) the feature distributions of 5 randomly selected novel classes from CIFAR-FS by our method, where each color represents a distinct category of features.

This paper introduces a novel algorithm using a dual-faceted knowledge distillation strategy aimed at enhancing the feature extraction capabilities of lightweight student models by learning from a more complex teacher model. To evaluate our method’s effectiveness, we applied the t-SNE [50] visualization technique to features extracted by the student model from test images. We selected five categories from the MiniImageNet and CIFAR-FS novel class datasets, randomly choosing 200 image samples per category. The t-SNE visualization results are presented in Figure 6, which includes both the baseline and our method. 

The t-SNE visualizations illustrate that our method achieves a more distinct separation of feature points across different categories compared to the baseline, which relies solely on cross-entropy loss and pre-training on base class datasets. Specifically, the visualization indicates that our method produces feature points that are more tightly clustered within the same category and more widely spaced between different categories. For instance, the overlap of red and green points is notably less in our method compared to the baseline on MiniImageNet. On CIFAR-FS, the feature points pertaining to our method are significantly more clustered, with a greater separation between clusters of different colors, suggesting an enhanced feature distinction capability. This visual comparison underscores our method’s enhanced capability in extracting distinctive features, thereby significantly advancing the performance in FSC tasks.

## 5. Conclusions

This study presents a novel approach to enhancing few-shot image classification in lightweight models that is particularly suited for scenarios with limited storage and computational resources, such as mobile and embedded devices. Our key contribution is the introduction of a dual-faceted knowledge distillation strategy, effectively improving the performance of lightweight models. This strategy combines output-based and intermediate feature-based distillation techniques, augmented by a novel feature error distribution calibration method. This calibration addresses the limitations of traditional mean squared error functions in knowledge distillation, ensuring a closer approximation to the teacher model’s feature distribution. Furthermore, we provide theoretical proof within the paper to substantiate this approach. This advancement not only enhances the understanding of knowledge distillation processes but also offers a robust framework for future research in this area.

Experiments conducted on multiple datasets, including MiniImageNet, demonstrate the superior performance of our method. Through rigorous ablation studies and comparisons with classical knowledge distillation algorithms, we have validated the enhanced base class classification and feature extraction capabilities of lightweight models using our approach. Notably, our method not only outperforms several established knowledge distillation algorithms but also surpasses current state-of-the-art methods based on lightweight models in few-shot image classification tasks.

In conclusion, the proposed model compression technique, leveraging a dual-faceted knowledge distillation strategy, offers a significant advancement in the field of few-shot learning for lightweight models. This advancement has potential applications in various real-world scenarios where computational efficiency and model size are critical considerations.

## Figures and Tables

**Table 1:** Comparative classification accuracy (%) of baseline and our proposed method.

Method	Backbone	MiniImageNet		CIFAR-FS		CUB	
		1-Shot	5-Shot	1-Shot	5-Shot	1-Shot	5-Shot
Baseline	Conv4-64	53.36	70.51	63.83	78.14	64.77	79.17
MC	Conv4-64	57.99	73.30	67.02	80.98	69.92	85.27

**Table 2:** Comparative classification accuracy (%) of KD-Plain and KD-Improved methods.

Dataset	Backbone	Setting	KD-Plain	KD-Improved
MiniImageNet	Conv4-64	1-shot	57.01	57.99 (0.98 ↑)
	Conv4-64	5-shot	72.69	73.30 (0.61 ↑)
CIFAR-FS	Conv4-64	1-shot	65.98	67.02 (1.04 ↑)
	Conv4-64	5-shot	80.02	80.98 (0.96 ↑)
CUB	Conv4-64	1-shot	68.87	69.92 (1.05 ↑)
	Conv4-64	5-shot	84.24	85.27 (1.03 ↑)

**Table 3:** Comparison of the proposed knowledge distillation method with classical approaches.

Method	Backbone	MiniImageNet		CIFAR-FS		CUB	
		1-Shot	5-Shot	1-Shot	5-Shot	1-Shot	5-Shot
HKD [22]	Conv4-64F	57.33 ± 0.41	72.42 ± 0.34	66.27 ± 0.93	80.35 ± 0.69	68.28 ± 0.50	84.07 ± 0.28
FitNet [23]	Conv4-64F	57.43 ± 0.45	72.26 ± 0.34	66.40 ± 0.91	80.44 ± 0.67	68.45 ± 0.48	83.63 ± 0.29
AT [24]	Conv4-64F	57.86 ± 0.48	72.79 ± 0.35	66.75 ± 0.92	80.72 ± 0.68	67.90 ± 0.48	83.53 ± 0.30
SP [25]	Conv4-64F	57.26 ± 0.45	72.61 ± 0.35	66.72 ± 0.92	80.68 ± 0.69	68.37 ± 0.47	84.05 ± 0.29
RKD [26]	Conv4-64F	57.14 ± 0.45	72.89 ± 0.35	66.73 ± 0.91	80.70 ± 0.68	68.68 ± 0.47	84.03 ± 0.29
PKT [27]	Conv4-64F	57.61 ± 0.48	72.91 ± 0.31	66.84 ± 0.92	80.70 ± 0.67	68.13 ± 0.47	84.02 ± 0.29
NST [28]	Conv4-64F	57.82 ± 0.48	72.66 ± 0.35	66.83 ± 0.93	80.72 ± 0.68	68.07 ± 0.48	84.02 ± 0.29
Ours	Conv4-64F	57.99 ± 0.44	73.30 ± 0.36	67.02 ± 0.92	80.98 ± 0.69	69.92 ± 0.46	85.27 ± 0.28

**Table 4:** Comparison of classification results (%) on MiniImageNet, CIFAR-FS, and CUB.

Method	Backbone	MiniImageNet		CIFAR-FS		CUB	
		1-Shot	5-Shot	1-Shot	5-Shot	1-Shot	5-Shot
Meta-learning paradigms							
MAML [12]	Conv4-64F	48.70 ± 1.75	63.11 ± 0.92	58.90 ± 1.90	71.50 ± 1.00	55.92 ± 0.95	72.09 ± 0.76
Prototypical [39]	Conv4-64F	49.42 ± 0.78	68.20 ± 0.66	51.31 ± 0.91	70.77 ± 0.69	-	-
Relational [40]	Conv4-64F	50.44 ± 0.82	65.32 ± 0.70	49.42 ± 0.78	68.20 ± 0.66	51.31 ± 0.91	70.77 ± 0.69
MetaOpt SVM [41]	Conv4-64F	52.87 ± 0.57	68.76 ± 0.48	-	-	62.45 ± 0.98	76.11 ± 0.69
PN+rot [14]	Conv4-64F	53.63 ± 0.43	71.70 ± 0.36	-	-	-	-
CovaMNet [42]	Conv4-64F	51.19 ± 0.76	67.65 ± 0.63	-	-	52.42 ± 0.76	63.76 ± 0.64
DN4 [43]	Conv4-64F	51.24 ± 0.74	71.02 ± 0.64	-	-	46.84 ± 0.81	74.92 ± 0.64
MeTAL [44]	Conv4-64F	52.63 ± 0.37	70.52 ± 0.29	-	-	-	-
PL [45]	Conv4-64F	48.00 ± 0.24	67.14 ± 0.23	-	-	60.11 ± 0.26	79.07 ± 0.25
LLDGP [46]	Conv4-64F	-	-	64.17 ± 0.31	78.42 ± 0.26	-	-
PSST [47]	Conv4-64F	-	-	64.37 ± 0.33	80.42 ± 0.32	-	-
HGNN [48]	Conv4-64F	55.63 ± 0.20	72.48 ± 0.16	-	-	69.02 ± 0.22	83.20 ± 0.15
ProtoNet+norm [15]	Conv4-64F	50.29 ± 0.41	67.13 ± 0.34	-	-	-	-
MC [16]	Conv4-64F	49.64 ± 0.83	65.67 ± 0.70	-	-	-	-
Transfer learning paradigms							
Baseline++ [17]	Conv4-64F	48.24 ± 0.75	66.43 ± 0.63	-	-	60.53 ± 0.83	79.34 ± 0.61
Neg-Cosine [24]	Conv4-64F	52.84 ± 0.76	70.41 ± 0.66	-	-	-	-
RFS-distill [29]	Conv4-64F	47.71	65.40	-	-	-	-
SKD [30]	Conv4-64F	48.14	66.36	-	-	-	-
CGCS [49]	Conv4-64F	55.53 ± 0.20	72.12 ± 0.16	-	-	-	-
Ours	Conv4-64F	57.99 ± 0.44	73.30 ± 0.36	67.02 ± 0.92	80.98 ± 0.69	69.92 ± 0.46	85.27 ± 0.28

- Indicates that the method described in the literature was not evaluated on certain datasets.

## Data Availability

The datasets used in our study are publicly available datasets in the field of few-shot learning.
